# Exploring the Antitumor Mechanism of High-Dose Cytarabine through the Metabolic Perturbations of Ribonucleotide and Deoxyribonucleotide in Human Promyelocytic Leukemia HL-60 Cells

**DOI:** 10.3390/molecules22030499

**Published:** 2017-03-21

**Authors:** Zheng Li, Jian-Ru Guo, Qian-Qian Chen, Cai-Yun Wang, Wei-Jia Zhang, Mei-Cun Yao, Wei Zhang

**Affiliations:** 1 State Key Laboratory of Quality Research in Chinese Medicine, Macau Institute for Applied Research in Medicine and Health, Macau University of Science and Technology, Taipa, Macau, China; lizhengcpu@163.com (Z.L.); guojianrumust@gmail.com (J.-R.G.); qqchen@must.edu.mo (Q.-Q.C.); Cywang@must.edu.mo (C.-Y.W.); 2 School of Pharmaceutical Sciences, Sun Yat-Sen University, Guang Zhou 510006, China; vega0313@126.com (W.-J.Z.); yaomeicun@gmail.com (M.-C.Y.)

**Keywords:** high-dose Ara-C, mechanism, LC-MS, ribonucleotide, deoxyribonucleotide, perturbation

## Abstract

Despite the apparent clinical benefits of high-dose cytarabine (Ara-C) over lower dose Ara-C in acute myeloid leukemia (AML) therapy, the mechanism behind high-dose Ara-C therapy remains uncertain. In this study, a LC-MS-based method was carried out to investigate the metabolic alteration of ribonucleotide and deoxyribonucleotide in human promyelocytic leukemia cells (HL-60) after treatment with Ara-C to reveal its antitumor mechanism. The metabolic results revealed that four nucleotides (ATP, ADP, CDP, and dCTP) could be used as potential biomarkers indicating the benefit of high-dose Ara-C over lower dose Ara-C treatment. Combining metabolic perturbation and cell cycle analysis, we conjectured that, apart from the acknowledged mechanism of Ara-C on tumor inhibition, high-dose Ara-C could present a specific action pathway. It was suggested that the pronounced rise in AMP/ATP ratio induced by high-dose Ara-C can trigger AMP-activated protein kinase (AMPK) and subsequently Forkhead Box, class O (FoxO), to promote cell cycle arrest. Moreover, the significant decrease in CDP pool induced by high-dose Ara-C might further accelerate the reduction of dCTP, which then aggravates DNA synthesis disturbance. As a result, all of these alterations led to heightened tumor inhibition. This study provides new insight in the investigation of potential mechanisms in the clinical benefits of high-dose Ara-C in therapy for AML.

## 1. Introduction

Cytarabine (cytosine arabinoside, Ara-C) is a nucleotide-analog chemotherapeutic drug used alone or in combination with other antineoplastic drugs to treat different forms of leukemia. Multiple clinical trials have demonstrated complete remission (CR) rates of 50%–60% and overall survival rates of 30%–40% among acute myeloid leukemia (AML) patients receiving Ara-C-based therapy [1,2,3]. Moreover, high-dose Ara-C-based therapy has the highest antileukemic efficacy of all currently used therapies in the treatment of AML [4,5,6,7].

**Figure 1:**
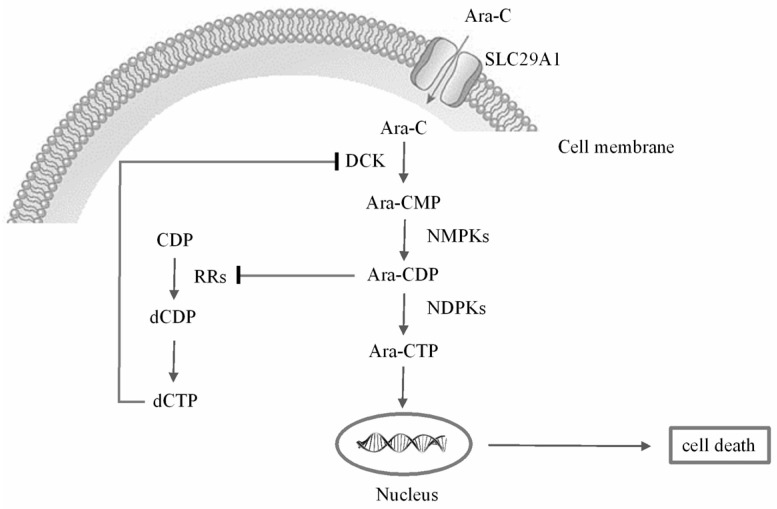
Schematic description of Ara-C transport and metabolism.

The anti-leukemia effect of Ara-C is the result of active uptake into target cells and the subsequent metabolism of Ara-C into its active metabolite. As shown in Figure 1, Ara-C is transported into cells mainly via nucleoside transporters including solute carrier family 29 member 1 (SLC29A1). Subsequently, intracellular Ara-C is phosphorylated to Ara-C monophosphate (Ara-CMP) by deoxy-cytidine kinase (dCK) [8,9], and then to Ara-C diphosphate (Ara-CDP) by cytidine monophosphate kinase 1 (CMPK1), and eventually to its active form Ara-C triphosphate (Ara-CTP) by several nucleoside diphosphate kinases (NDPKs) [10,11]. Ara-CTP competes with deoxycytidine triphosphate (dCTP) for incorporation into DNA and consequently causes cell death by interfering with DNA and RNA synthesis [11,12,13]. Several feedback mechanisms influence the metabolism of Ara-C; for example, deoxycytidine triphosphate (dCTP) is a potent feedback inhibitor of dCK [14], while intracellular dCTP pools are regulated by ribonucleotide reductase holoenzyme (RRs) [15].

Ara-CTP constitutes the main cytotoxic component, while dCK activity comprises the rate-limiting step for Ara-CTP formation. However, the concentration achieved by high-dose Ara-C treatment exceeds by far the concentration for saturation of dCK. Thus, the ability to synthesize intracellular Ara-CTP is saturable [16,17]. Despite the apparent clinical benefits of high-dose Ara-C over lower dose Ara-C therapy, it is unlikely that the clinical benefits of high-dose Ara-C are gained from an increased peak intracellular Ara-CTP level. The mechanism behind high-dose Ara-C therapy remains uncertain. Improvements in AML treatment could be achieved by better understanding the mechanism of Ara-C.

It is well known that the action of nucleoside analogues against cancer can disturb endogenous ribonucleotide (RN) and deoxyribonucleotide (dRN) pool sizes, which play essential roles in a broad range of key cellular functions. In contrast, the action can also be affected by RN and dRN pool sizes [18,19]. An unbalanced change of RN and dRN pool sizes can lead to genetic abnormalities or cell death in mammalian cells [20]. In order to understand the exact mechanism of action of high-dose Ara-C, it is critical to elucidate the disturbances of Ara-C treatment on RN and dRN pool sizes.

Considering the importance of RNs and dRNs as the most affected metabolites during Ara-C treatment, a LC-MS method was used to investigate the metabolic alteration of nucleotides in human promyelocytic leukemia cells (HL-60) after treatment with different doses of Ara-C to reveal its antitumor mechanism. The obtained LC-MS data were analyzed to determine potential biomarkers and related metabolic pathways. Furthermore, cell cycle analysis was carried out to assist metabolic illustration.

## 2. Results and Discussion

### 2.1. MTT Assay

**Figure 2:**
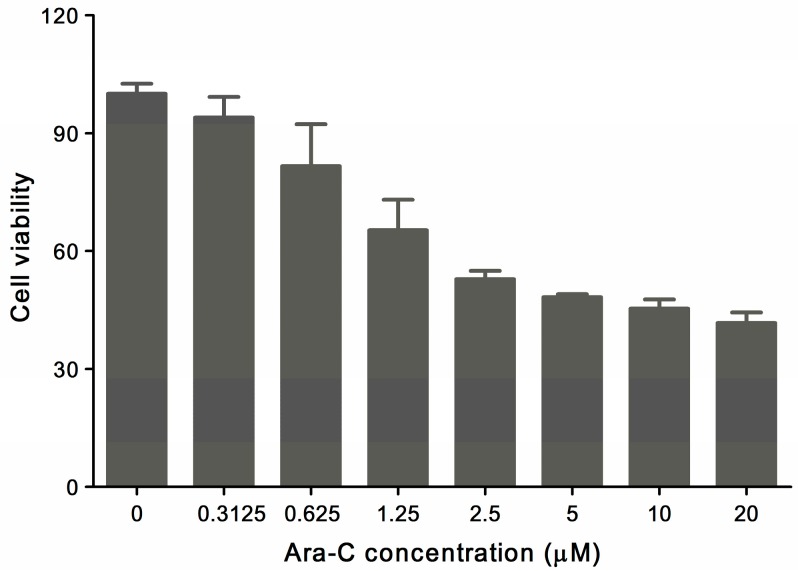
The cytotoxicity of Ara-C on HL-60 cells at different concentrations after 24 h treatment (*n* = 6).

The antiproliferative effect of Ara-C on HL-60 cells was investigated with MTT assay to assess the selected drug concentrations for the following studies. As shown in Figure 2, the toxicity of Ara-C was obvious when drug concentration was more than 0.625 μM. The HL-60 cell viability decreased in a dose-dependent manner, and only 50% of cells were viable when concentration increased to 2.5 μM and the calculated IC_50_ of Ara-C was close to 2.5 μM. It is noteworthy that, when HL-60 cells were treated with Ara-C at the range between 2.5 and 20 μM for 24 h, its viability was approximately 50%, which presented small variation.

### 2.2. Cell Cycle Arrest Induced by Ara-C

**Figure 3:**
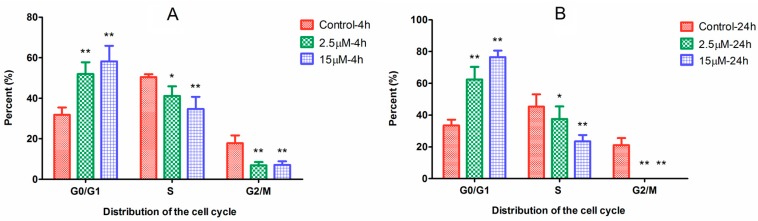
Effects of different dose Ara-C on cell cycle arrest in HL-60 cells at different times. (**A**) 4 h; (**B**) 24 h. *p*-Value of less than 0.05 (* *p* < 0.05, ** *p* < 0.01, compared with the control group) are considered significant.

The active metabolite of Ara-C can incorporate into DNA to inhibit DNA synthesis. In order to investigate the effect of difference dosage Ara-C on the cell cycle phase distribution in HL-60 cells, the cell cycle analysis was carried out. After drug treatment, the cell cycle distribution significantly changed (Figure 3). Specifically, treatments of HL-60 cells with Ara-C at 2.5 and 15 µM for 4 h resulted in significant increases in the percentage of cells in G0/G1 phase from 31.8% ± 3.5% to 50.6% ± 5.6% and 57.1% ± 8.9%, respectively, both *p* < 0.01. There was a progressive increase in G0/G1 phase arrest from 4 to 24 h. Finally, treatment with Ara-C at 2.5 and 15 µM for 24 h induced cell cycle arrest in G0/G1 phase from 33.5 ± 3.6% to 63.1% ± 9.5% and 75.5% ± 4.2%, respectively, both *p* < 0.01. These results indicated that Ara-C could block HL-60 cells in G0/G1 phase to inhibit cell growth or cancer progression. Furthermore, high-dose Ara-C (15 µM) induces more severe arrest in G0/G1 phase than does low-dose Ara-C (2.5 µM).

### 2.3. Metabolic Alterations Following Treatment with Ara-C

Table 1 and Table 2 show the general properties and differences in RN and dRN pool sizes of HL-60 cells before and after incubation with Ara-C at different doses and time periods. The routine-dose (2.5 µM) and high-dose (15 µM) groups shared similar numbers of survival cells, which were approximately half of the control groups, indicating that the two doses of Ara-C exerted equal cytotoxicity. For RN pool sizes, after 4 and 24 h incubation, no consistent significant increase was observed at routine-dose Ara-C. However, we observed significant decreases in ATP, ADP, CDP, GDP, UDP, and UMP levels after high-dose Ara-C treatment. Moreover, the consistent significant increases in AMP/ATP ratio (0.003 ± 0.0 vs. 0.005 ± 0 * at 4 h, 0.011 ± 0.002 vs. 0.034 ± 0.005 ** at 24 h) due to the decrease in ATP were observed after 4 and 24 h incubation of high-dose Ara-C.

For dRN pool sizes, after 4 and 24 h incubation, dATP and dTMP levels demonstrated significant increases in routine-dose Ara-C groups, while dCTP, dCDP, and dGDP levels demonstrated significant decreases in high-dose Ara-C groups. As Ara-C exerts efficacy by competing with dCTP for incorporation into DNA and inhibiting RRs through its active metabolite Ara-CTP, the perturbation of dCDP and dCTP levels would be the most pronounced. The depletions of dCDP and dCTP in high-dose Ara-C groups were more evident than that in routine-dose Ara-C groups, which suggested that high-dose Ara-C therapy could more severely and persistently perturb cellular metabolism than routine-dose Ara-C therapy. The severely perturbed cellular metabolism, especially dCDP and dCTP metabolism, could be one potential mechanism of the clinical benefits gained from high-dose Ara-C therapy.

### 2.4. Metabolic Biomarkers and Pathway Analysis

**Figure 4:**
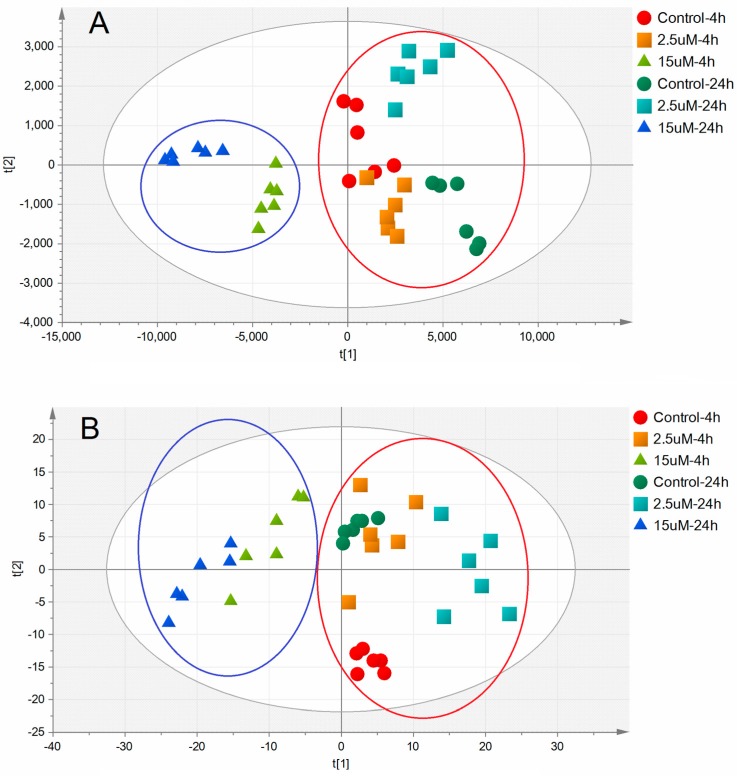
Multivariate analytical results of extracted metabolites in HL-60 cells with or without Ara-C treatment. (**A**) 2D PLS-DA score plot of NPs data; (**B**) 2D PLS-DA score plot of dNP data. Scores t[1] and t[2] created by the algorithm of PLS-DA, are new variables summarizing input variables.

Figure 4 shows the PLS-DA score plots, representing the distribution between the control groups and Ara-C groups with two principle components. An obvious separation was observed between high-dose Ara-C groups and control groups, indicating that the RN and dRN metabolic profiles changed significantly as a result of toxicity, while routine-dose Ara-C groups were not distinguished from control groups. Statistics analysis showed that high-dose Ara-C remarkably altered the profiles of 6 RNs (ATP, ADP, CDP, CTP, GTP, and UTP) and 4 dRNs (dATP, dCTP, dGTP, and dTTP) with VIP > 1. Ultimately, the differences among groups were evaluated for individual metabolites by using an independent-samples *t*-test (*p* < 0.01) combined with a VIP value generated in PLS-DA (VIP > 1) and only four metabolites (ATP, ADP, CDP, and dCTP) were eventually selected as potential biomarkers.

**Figure 5:**
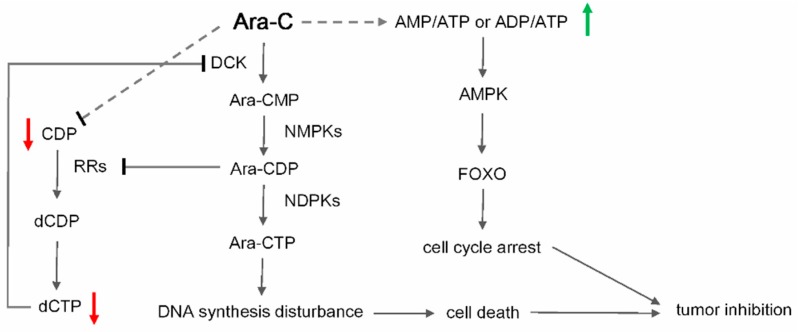
Hypothetic scheme of the antitumor mechanism of high-dose Ara-C.

To summarize the potential biomarkers and pathways disturbed by high-dose Ara-C to reveal possible action mechanisms, a metabolic pathway scheme was given in Figure 5. As a whole, apart from the acknowledged mechanism of Ara-C on tumor inhibition, which was discussed above, high-dose Ara-C could present a specific action pathway.

AMP-activated protein kinase (AMPK) is one of the key regulators of cellular metabolism, which can be regulated by the cellular AMP/ATP ratio [21]. The consistent significant increases in AMP/ATP ratio would active AMPK. Forkhead Box class O (FoxO) proteins are a subfamily of transcription factors involved in tumor suppression, the regulation of energy metabolism, cell cycle, and apoptosis [22,23,24]. The transcriptional activity of FoxO is further modulated by AMPK. Furthermore, recent studies have revealed that the FoxO family, particularly FoxO3a, has emerged as playing an important role in the cell cycle arrest and apoptosis of hematopoietic cells [25,26,27]. The activation of FoxO3a could override growth factor-independent survival and induce cell cycle arrest and apoptosis.

Thus, we conjectured that the significant decrease in the ATP pool induced by high-dose Ara-C resulted in a pronounced AMP/ATP ratio rise, which triggered AMPK and subsequently FoxO to promote cell cycle arrest. This conjecture was consistent with the cell cycle observation of more severe cell cycle arrest in G0/G1 phase in the high-dose Ara-C group compared with the routine-dose Ara-C group. Moreover, the significant decrease in the CDP pool induced by high-dose Ara-C further accelerated the reduction of dCTP pool, which then aggravated DNA synthesis disturbance. As a result, all these alterations in metabolism led to heightened tumor inhibition. The hypothesis could partly explain the mechanism behind the clinical benefits from high-dose Ara-C in therapy for AML.

## 3. Materials and Methods

### 3.1. Chemicals and Reagents

Cytarabine for injection used in this study was purchased from Actavis Italy S.p.A. Human acute promyelocytic leukemia cell line HL-60 was supplied by American Type Culture Collection (ATCC) (Rockville, MD, USA). Phosphate buffer saline (PBS), RPMI medium 1640, 0.25% Trypsin-EDTA solution, penicillin-streptomycin solution, and fetal bovine serum (FBS) were obtained from GIBCO Invitrogen Co. (Carlsbad, CA, USA). A Cell Cycle Analysis Kit was purchased from Signal-way Antibody Co., Ltd. (College Park, MD, USA). Trichloroacetic acid (TCA), hexylamine (HA), diethylamine (DEA), trioctylamine, 1,1,2-trichlorotrifluoroethane and stable isotope labeled adenosine-^13^C_10_^15^N_5_-triphosphate (ATP^13^C^15^N) were purchased from Sigma-Aldrich Chemical Co. (St. Louis, MO, USA). LC-MS grade methanol, acetonitrile, and acetic acid were purchased from Anaqua Chemical Supply Co. (Houston, TX, USA). Ultra-pure water was obtained from the Milli-Q Gradient Water System (Millipore Corporation, Billerica, MA, USA).

### 3.2. Cell Culture

The HL-60 cells were seeded in 100 mm × 20 mm dishes (LabServ, Thermo Fisher Scientific, Beijing, China) and cultured in RPMI Medium 1640 supplemented with 10% FBS, 100 UI/mL penicillin, and 100 μg/mL streptomycin in humidified air at 37 °C with 5% CO_2_. After overnight culture, cells were divided into two groups: a control group and an experimental group. Cells of the control group were incubated in a medium only. Cells of the routine-dose group were incubated for 4 and 24 h with 2.5 µM Ara-C, which is almost equivalent to the routine-dose regimens with 0.5–1 g/m^2^ Ara-C plasma concentrations. Cells of the high-dose group were incubated with 15 µM Ara-C, which is almost equivalent to the plasma concentrations (2–3 g/m^2^ Ara-C) achieved during high-dose Ara-C regimens [6].

### 3.3. MTT Assay

The cytotoxicity of cell growth by Ara-C was investigated via the MTT assay. HL-60 cells were seeded in 96-well plate (LabServ, Thermo Fisher Scientific, Beijing, China) at a density of 2 × 10^4^ per well. After incubation, they were treated with Ara-C at different concentrations for 24 and 48 h. MTT solution (final concentration of 0.5 mg/mL in medium) was added to each well and incubated further for 4 h. The medium was removed and 100 μL of DMSO was added to each well to dissolve the purple crystals of formazan. Absorbance was measured at 570 nm with a microplate UV-Vis spectrophotometer (Infinite M200 PRO, Tecan Auatria); reference wavelength was 650 nm. IC_50_ values of Ara-C were calculated by GraphPad Prism Software Inc. (San Diego, CA, USA).

### 3.4. Cell Cycle Analysis

Cells were seeded at 4 × 10^5^ cells/well in 6-well culture plates in duplicate, and incubated with Ara-C at 2.5 (routine-dose) and 15 µM (high-dose) for 4 and 24 h. They were then harvested and fixed in 70% (*v*/*v*) cold ethanol overnight at 4 °C. The fixed cells were collected by centrifugation and re-suspended in PBS. Subsequently, the re-suspended cells were incubated with 5 mg/mL propidium iodide (Sigma-Aldrich) and 10 mg/mL RNase A (Sigma-Aldrich) at room temperature for 30 min in the dark. The cells were then analyzed on a flow cytometer (Muse^TM^ cell analyzer, Merck Millipore, Darmstadt, Germany). Finally, the percentages of cells in different phases (G0/G1, S and G2/M) were calculated using Modfit software (Verity Software House, Topsham, ME, USA).

### 3.5. Sample Preparation

After removal of the culture medium, cells were washed once with precooled PBS and then treated with trypsin. Each digested sample was re-suspended in 10 mL of PBS, followed by centrifugation (5 min; 1500× *g*; 4 °C), and the pellets were frozen in liquid nitrogen and stored at −80 °C for further analysis. Extraction was performed by addition of 150 µL of 15% TCA containing 7.5 µL of 20.0 µM ATP^13^C^15^N as an internal standard and placed on ice for 10 min. After centrifugation at 13,500 rpm for 15 min in a cold room, the acidic supernatant was separated and neutralized twice with 100 µL of a mixture of trioctylamine and 1,1,2-trichlorotrifluoroethane (45:55, *v*/*v*). Samples were stored at −80 °C until analysis within two days.

### 3.6. LC-MS/MS Analysis

LC-MS/MS analysis was performed on a Thermo Fisher TSQ LC-MS/MS system (Thermo Fisher Scientific Co., San Jose, CA, USA) consisting of an Accela Autosampler, an Accela pump, and a Quantum Access triple quadrupole mass spectrometer. Chromatography was performed on an XTerra-MS C18 column (150 mm × 2.1 mm, 3.5 µm) (Waters Corp., Milford, MA, USA). The column was maintained at 35 °C and the flow rate was 0.3 mL/min. The mobile phase was composed of Solvent A (5 mM HA-0.5% DEA in water, pH adjusted to 10 with acetic acid) and Solvent B (50% acetonitrile in water). The column was eluted with a linear gradient system: 0–15 min, 100%–88% A; 15–35 min, 88%–72% A; 35–45 min, 72%–45% A; 45–50 min, 45%–100% A; 50–60 min, 100%–100% A. For all RN and dRN, the following optimized parameters were obtained. The sheath gas pressure reached 40 psi. The ionspray voltage was set at 3000 V for negative mode and 4000 V for positive mode at a temperature of 350 °C and an auxiliary gas pressure of 15 psi. Quantification was performed using multiple reactions monitoring (MRM) as previously published [28]. Data acquisition was performed with the Xcalibur software version 2.0.7 (San Jose, CA, USA), and data processing using the Thermo LCquan 2.5.6 data analysis program (Thermo Fischer, San Jose, CA, USA).

### 3.7. Statistics Analysis and Potential Metabolic Biomarkers Determination

Results are mean ± SD and evaluated using the independent-samples Student’s *t* test. *p* < 0.05 was considered to be significant and *p* < 0.01 to be very significant.

SIMCA-P software (V13.0, Umetrics, Umea, Sweden) was used to conduct partial least squares-discriminant analysis (PLS-DA) model for pattern recognition. The PLS-DA model was used to separate samples into two blocks and obtain clear discrimination between the control- and Ara-C-treated (2.5 and 15 µM) groups. Variables that made significant contributions to discriminating between groups were considered to be potential biomarkers. The variable importance in the projection (VIP) values was used to select biomarkers. Variables with a VIP exceeding 1 showed a higher than average influence on the classification.

Ultimately, the differences among samples were evaluated for individual metabolites by using an independent-samples *t*-test and a VIP value generated by SIMCA-P in the PLS-DA model of more than 1.0. These metabolites were eventually selected as potential biomarkers.

## 4. Conclusions

In this study, a LC-MS-based method was used to investigate the metabolic alteration of ribonucleotide and deoxyribonucleotide in HL-60 cells after treatment with different doses of Ara-C to reveal its antitumor mechanism. The statistic results revealed that four nucleotides (ATP, ADP, CDP, and dCTP) could be used as potential metabolic biomarkers. Combining metabolic alterations and biological results, we conjectured that, apart from the acknowledged mechanism of Ara-C on tumor inhibition, high-dose Ara-C could present a specific action pathway. It was suggested that the pronounced rise in AMP/ATP ratio due to the significant reduction of ATP induced by high-dose Ara-C triggered AMPK and subsequently FoxO to promote cell cycle arrest. Moreover, the significant decrease in CDP pool induced by high-dose Ara-C further accelerated the reduction of dCTP pool, which then aggravated DNA synthesis disturbance. As a result, all these alterations led to heightened tumor inhibition. This study provides new insight in the investigation of the potential mechanism in the clinical benefits of high-dose Ara-C in therapy for AML, which can assist in its improvement and applications.

## Figures and Tables

**Table 1:** Levels of RNs in HL-60 cells before and after incubation with Ara-C (pmol/10^6^ cell).

	Control-4 h	2.5 μM-4 h	15 μM-4 h	Control-24 h	2.5 μΜ-24 h	15 μΜ-24 h
**ATP**	14,558.32 ± 685.79	15,117.47 ± 498.53	9428.09 ± 508.33 **	17,822.85 ± 441.51	17,085.81 ± 1050.99	5934.37 ± 507.32 **
**ADP**	1002.55 ± 217.5	768.84 ± 99.63	489.81 ± 72.97 **	2551.64 ± 288.24	3239.08 ± 804.21	738.05 ± 153.52 **
**AMP**	52.35 ± 21.93	54.4 ± 11.51	46.33 ± 8.71	190.5 ± 36.57	349.7 ± 84.19 *	195.34 ± 62.2
**CTP**	1686.13 ± 369.04	2675.99 ± 507.97 **	2126.18 ± 310.6	2001.85 ± 349.47	2308.94 ± 620.87	1187.43 ± 156.51 **
**CDP**	136.78 ± 46.11	133.47 ± 24.9	102.6 ± 29.83 *	362.11 ± 83.62	481.79 ± 210.33	115.73 ± 35.45 **
**CMP**	46.67 ± 12.03	54.39 ± 18.45	49.57 ± 15.37	66.44 ± 10.55	181.33 ± 105.04 *	111.8 ± 51.61
**GTP**	2169.34 ± 589.95	2923.08 ± 951.26	1911.77 ± 262.31	3453.18 ± 213.16	2297.68 ± 689.24 *	1301.35 ± 532.45 **
**GDP**	252 ± 35.18	243 ± 27.63	192.55 ± 20.4	607.68 ± 76.64	842.26 ± 126.22 **	331.8 ± 41.29 **
**GMP**	5.54 ± 1.74	3.7 ± 0.83	3.17 ± 0.98	18.39 ± 3.16	42.2 ± 18.64 *	17.79 ± 8.25
**UTP**	4907.34 ± 1309.09	6819.2 ± 935.39 *	4269.28 ± 446.38	9065.78 ± 1205.95	4986.46 ± 685.95 **	1817.11 ± 259.88 **
**UDP**	257.54 ± 100.54	180.96 ± 46.09	135.55 ± 24.21 *	674.1 ± 103.75	671.86 ± 266.56	179.18 ± 64.47 **
**UMP**	6.83 ± 3.21	5.22 ± 1.55	4.19 ± 1.32	20.43 ± 4.2	27.28 ± 9.79	9.61 ± 4.56 **
**AMP/ATP**	0.003 ± 0.0	0.003 ± 0	0.005 ± 0 *	0.011 ± 0.002	0.02 ± 0.006	0.034 ± 0.005 **

Each data point is an average of two independent experiments (each performed in triplicate) and is reported as mean ± SD. (* *p* < 0.05, ** *p* < 0.01, compared with the control group).

**Table 2:** Levels of dRNs in HL-60 cells before and after incubation with Ara-C (pmol/10^6^ cell).

	Control-4 h	2.5 μM-4 h	15 μM-4 h	Control-24 h	2.5 μΜ-24 h	15 μΜ-24 h
**dATP**	6.77 ± 1.25	12.31 ± 3.14 **	9.22 ± 1.08 *	9.95 ± 1.15	14.81 ± 3.17 *	7.1 ± 1.37 **
**dADP**	0	0	0	0.015 ± 0.006	0.048 ± 0.042	0.003 ± 0.001 **
**dAMP**	0	0	0	0	0.01 ± 0	0
**dCTP**	22.39 ± 8.85	20.75 ± 3.42	10.63 ± 1.73 **	16.55 ± 2.23	34.55 ± 5.77 **	7.83 ± 1.08 **
**dCDP**	0.009 ± 0	0.004 ± 0.003 *	0 **	0.014 ± 0.006	0.063 ± 0.038 *	0 **
**dCMP**	0	0	0	0.016 ± 0.004	0.006 ± 0.005 *	0 **
**dGTP**	1.88 ± 0.8	3.68 ± 2.76	2.14 ± 1.24	5.33 ± 2.39	2.94 ± 2.11	0.66 ± 0.56 **
**dGDP**	0.12 ± 0.07	0.07 ± 0.04 *	0.05 ± 0.01 *	0.66 ± 0.06	0.66 ± 0.2	0.04 ± 0.03 **
**dGMP**	0.022 ± 0.021	0.103 ± 0.061 *	0.029 ± 0.011	0.168 ± 0.048	0.234 ± 0.111	0.047 ± 0.042 **
**dTTP**	25.54 ± 3.59	41.22 ± 6.19 **	32.76 ± 6.67	41.3 ± 3	50.92 ± 6.57	20.83 ± 5.18 **
**dTDP**	0.91 ± 0.29	0.99 ± 0.12	0.66 ± 0.15 *	1.18 ± 0.24	3.57 ± 0.61 **	1.15 ± 0.57
**dTMP**	0	0.011 ± 0.007 *	0.01 ± 0.006 *	0.012 ± 0.002	0.038 ± 0.02 **	0.008 ± 0.008

Each data point is an average of two independent experiments (each performed in triplicate) and is reported as mean ± SD. (* *p* < 0.05, ** *p* < 0.01, compared with the control group).
